# Intracranial Injury Following Nasogastric Tube Placement After Skull Base Surgery: A Case Report and Systematic Review

**DOI:** 10.7759/cureus.89085

**Published:** 2025-07-30

**Authors:** Gabrielle Wolter, Zain U Naqvi, Ali Jalali, Tran Locke, K Kelly Gallagher, Meha G Fox

**Affiliations:** 1 Department of Otolaryngology, Baylor College of Medicine, Houston, USA; 2 School of Medicine, Baylor College of Medicine, Houston, USA; 3 Department of Neurosurgery, Baylor College of Medicine, Houston, USA

**Keywords:** feeding tube complications, intracranial dobhoff tube, intracranial feeding tube, intracranial nasogastric tube, skull base surgery, transsphenoidal surgery complications

## Abstract

Inadvertent intracranial nasogastric tube placement is a recognized risk following skull base fracture, but prior skull base surgery also poses a significant and underrecognized risk for this potentially fatal complication. We report the case of a 75-year-old female admitted with *Clostridioides difficile* colitis, six months after endoscopic endonasal resection of a pituitary macroadenoma. A systematic review identified 10 prior cases of intracranial tube placement following skull base or sinonasal surgery, including nasotracheal and feeding tube insertions. Literature suggests that using small-bore flexible tubes and preserving anatomical barriers, such as the position of the middle turbinate and intact bony structures like the sphenoid sinus roof, may reduce risk. We aim to characterize the risk of iatrogenic intracranial tube placement through a systematic review and a representative case.

## Introduction

Nasoenteric tubes are commonly placed in the hospital setting for gastric decompression and administration of enteral feeds [1]. These procedures are generally straightforward, with minor complications, such as pain, epistaxis, or sinusitis, and rare but serious complications including aspiration pneumonia, pneumothorax, and esophageal perforation [2]. One catastrophic risk is intracranial misplacement. For this reason, trauma resulting in skull base fracture is a well-documented contraindication for the placement of a nasoenteric tube [1,3]. However, the risk presented by previous anterior skull base surgery is less well-known. Anterior skull base surgery, including endoscopic endonasal approaches, is commonly performed for the resection of sellar and extrasellar skull base pathology [4]. Reconstruction of resulting skull base defects is completed with the triplicate goal of sealing existing CSF leaks, eliminating dead space, and preventing future CSF leaks [5]. This is often accomplished with soft tissue or pliable dural substitutes only; bony reconstruction is rarely performed due to the complex anatomy of the region [6]. This defect can thus become an intracranial pathway if traumatized by blind instrumentation, such as nasoenteric tube placement. We present a case of a 75-year-old female who developed fatal tension pneumocephalus, a life-threatening accumulation of air in the cranial vault causing mass effect, and pontine hemorrhage after inadvertent intracranial nasogastric tube (NGT) placement six months following endoscopic endonasal resection of a pituitary macroadenoma. Additionally, we conducted a systematic review of the literature to characterize this rare but devastating complication better.

## Case presentation

A 75-year-old female with a past medical history of hypertension and hyperlipidemia presented to an outside hospital with acute-onset headache and somnolence. Brain MRI demonstrated pituitary macroadenoma complicated by apoplexy. She was transferred to a tertiary care academic center for surgical intervention and underwent urgent endoscopic endonasal transsphenoidal resection of the pituitary macroadenoma. After resection of the tumor, no CSF leak was appreciated. Notably, there was a relative lack of sellar bone due to tumor erosion extending from the clivus to the tuberculum sellae. The sellar defect was repaired with absorbable packing under the diaphragma followed by dural substitute placement in the epidural space. A layer of dural sealant was then applied over the sella. The middle turbinates were medialized after closure with the use of absorbable packing. The patient was discharged on postoperative day 5 after an uncomplicated inpatient recovery period.

**Figure 1:**
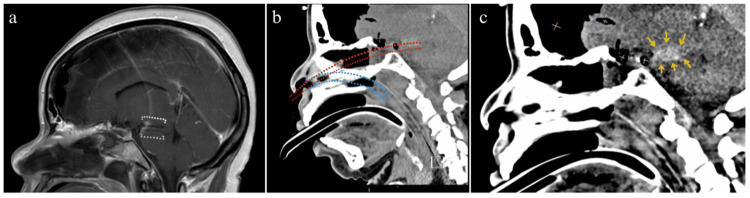
(a) MRI brain, sagittal view showing pontine hemorrhage (between dotted lines). (b) CT showing the path that the NGT travelled through the superior pons (red), and the planned NGT path (blue). (c) CT showing pontine hemorrhage (arrows), and pneumocephalus along the frontal convexities (yellow X) NGT, nasogastric tube.

Six months later, the patient presented to an outside hospital with *Clostridioides difficile* colitis. During her hospitalization, she reportedly became non-responsive, with CT of the head showing pneumocephalus. Specific details regarding her presentation at the outside hospital were not available for review upon transfer; therefore, information was gathered from the transferring team and collateral, including family members. She was transferred to the same tertiary care academic center where her pituitary macroadenoma was resected for further management of a suspected CSF leak. The patient was severely obtunded with a Glasgow Coma Scale (GCS) of 4 on continuous positive airway pressure upon arrival to the neurologic intensive care unit, requiring emergent intubation. Emergent burr hole and drain placement was performed by the neurosurgical team for evacuation of tension pneumocephalus. Once the patient was stabilized, additional history was elicited that an NGT was placed at the outside hospital after several unsuccessful attempts. It was shortly after placement of this feeding tube that the patient reportedly exhibited altered mental status for which she was placed on positive pressure ventilation, and subsequent CT head demonstrated the aforementioned pneumocephalus that prompted her transfer. Once this collateral information had been obtained, endoscopic evaluation at the tertiary facility showed a defect at the superior aspect of prior sellar repair with air and CSF expression seen with the application of pressure on the abdomen, most consistent with a traumatic injury. Notably, the NGT was in an appropriate position at the time of arrival at the tertiary institution (Figure 1). It is not known if the NGT was initially malpositioned and was repositioned correctly prior to arrival or if the injury was made during eventual correct placement due to a lack of outside hospital records. The patient’s neurologic exam demonstrated minimal upper extremity response to noxious stimuli with present corneal, gag, and cough reflexes. Once the patient was medically stabilized, she was taken to the operating room for skull base repair with the placement of a dural substitute in the epidural space and an overlying pedicled nasoseptal flap. Unfortunately, the patient’s neurological status remained poor with no significant improvement from prior exams. Serial imaging demonstrated evolving pontine hemorrhage. The patient was terminally extubated 18 days after the injury.

## Discussion

A systematic review of the literature regarding traumatic injury of the skull base following endoscopic endonasal skull base surgery was conducted in accordance with PRISMA guidelines. MEDLINE, EMBASE, and PubMed databases were queried for titles or abstracts with the following keywords: (“nasogastric tube” or “NGT” or “feeding tube” or “enteral tube” or “dobhoff tube” or “DHT” or “nasogastric feeding tube” or “nasoenteral tube” or “nasotracheal tube”) and (“intracranial” or “brain”) and (“placement” or “insertion”). Search strings and years included from each database can be found in Table 1.

**Table 1:** Database search strategy

Name of Database	Years Searched	Search Query
EMBASE	1957-2023	("nasogastric tube"/exp OR "nasogastric tube" OR ngt OR "feeding tube"/exp OR "feeding tube" OR "enteral tube" OR "dobhoff tube"/exp OR "dobhoff tube" OR dht OR "nasogastric feeding tube"/exp OR "nasogastric feeding tube" OR "nasoenteral tube"/exp OR "nasoenteral tube" OR "nasotracheal tube"/exp OR "nasotracheal tube") AND (intracranial OR "brain"/exp OR brain) AND (placement OR "insertion"/exp OR insertion)
MEDLINE	1946-2023	(("nasogastric tube" or NGT or "feeding tube" or "enteral tube" or "dobhoff tube" or DHT or "nasogastric feeding tube" or "nasoenteral tube" or "nasotracheal tube") and (intracranial or brain) and (placement or insertion)).mp
PubMed	1972-2023	("nasogastric tube" OR NGT OR "feeding tube" OR "enteral tube" OR "dobhoff tube" OR DHT OR "nasogastric feeding tube" OR "nasoenteral tube" OR "nasotracheal tube") AND (intracranial OR brain) AND (placement OR insertion)

**Figure 2:**
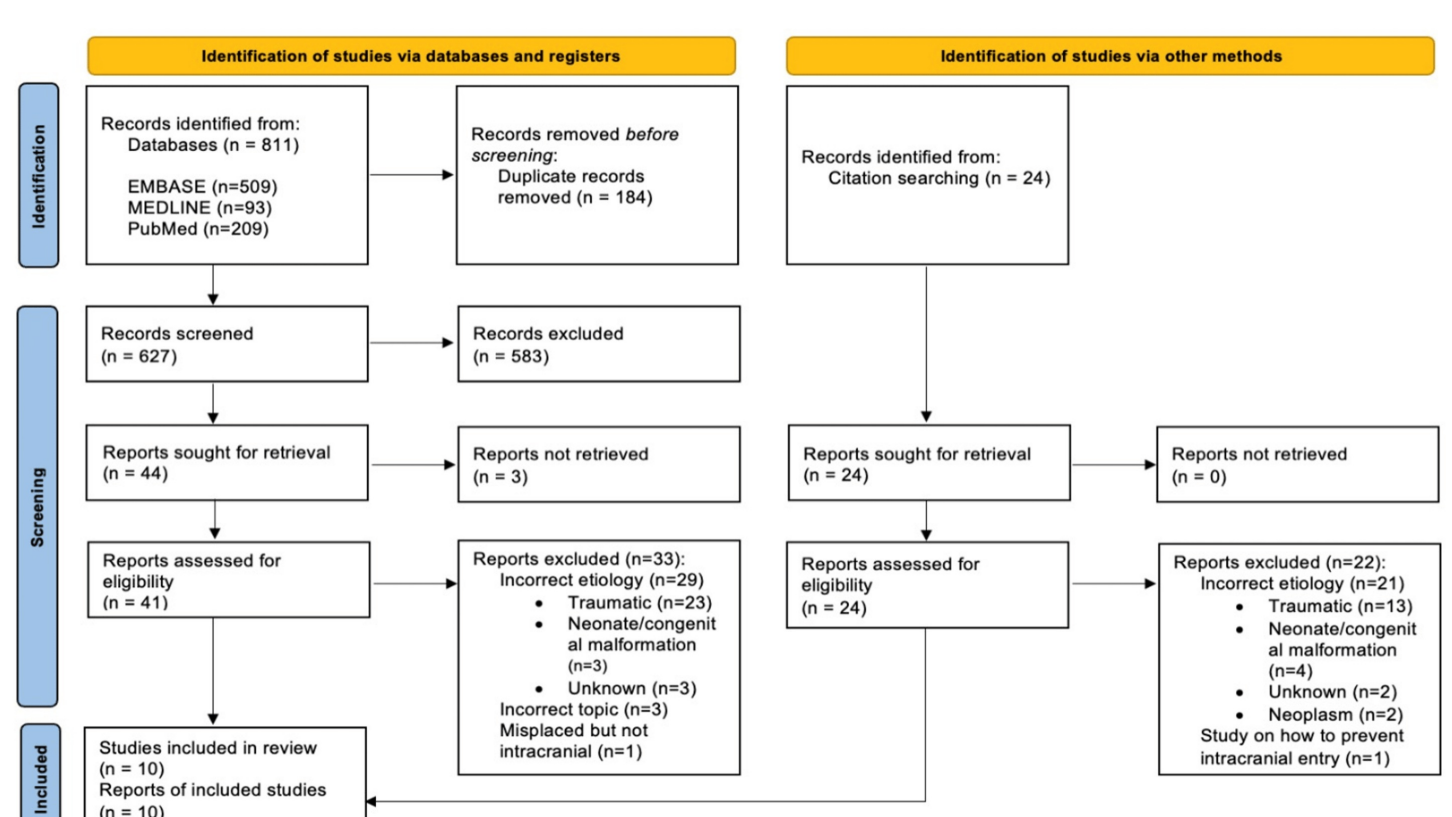
PRISMA flowchart PRISMA, Preferred Reporting Items for Systematic reviews and Meta-Analyses. Ref. [7].

A comprehensive search initially identified 811 records (EMBASE: 509, PubMed: 209, and MEDLINE: 93). After removal of 184 duplicates, 627 records were screened by two independent reviewers (GW, ZN). Of these, 583 were excluded based on title and abstract. Forty-four reports were sought for retrieval, with three reports not retrieved. Forty-one full-text articles were assessed for eligibility, with 33 being excluded. Citation searching of reference lists and suggested articles yielded an additional 24 records, from which two were included. In total, 10 studies met final inclusion criteria and were incorporated into the review (Figure 2). Including our institutional case, a total of 11 cases were analyzed to better characterize the risk of iatrogenic intracranial tube placement following skull base or sinonasal procedures. Articles were included if they were in English, described intracranial malposition of a tube (nasogastric, nasoenteral, or nasotracheal), and involved patients with a history of skull base or sinonasal procedures. Exclusion criteria included non-English language, extracranial tube malposition, or skull base defects due to trauma, congenital, infectious, or unknown causes. Data were systematically extracted by a single reviewer and included case details, tube type, timing of insertion relative to surgery, etiology of the skull base defect, insertion point, and clinical outcomes.

Eight cases occurred after endoscopic endonasal resection of sellar pathology, and two occurred after other craniofacial surgery (septoplasty, choanal atresia repair) [8-17]. Seven cases involved NGTs, and one each involved a nasotracheal suction, nasotracheal tube, and small-bore feeding tube [8-17]. Four patients died soon after the event, and four others experienced severe neurologic sequelae [8-13,15,16]. The length of time between surgery and intracranial insertion ranged from the day of surgery to six months after surgery. A summary of the data can be found in Table 2.

**Table 2:** Summary of included studies This table presents individual case reports of intracranial placement of nasally introduced tubes in patients with prior skull base pathology or surgery. It summarizes the surgical context, type of tube used, timing (postoperative day), presumed entry site, and resulting patient outcomes. NGT, nasogastric tube; POD, postoperative day; TSA, transsphenoidal approach; ICP, intracranial pressure; LOC, level of consciousness; TCD, transcranial Doppler; CTA, CT angiography. ^†^Not specified in the original case report.

First Author (Year)	Surgery	Case Summary	Tube	POD	Entry Site	Patient Outcome
Hande (1991) [8]	Revision TSA for partial resection of pituitary tumor	Developed meningitis with CT showing intracranial and pneumocephalus	NGT	†	†	Death 2 days after removal
Metheny 2002 [9]	TSA for resection of pituitary tumor	CSF leak POD17, underwent leak repair with recurrent leak POD6. Developed apnea and weakness, NGT placed in preparation for intubation. Post-intubation CT showed NG through sphenoid into right parietal lobe and lateral ventricle.	18F NGT	7	†	Further deterioration with CT scan showing R thalamic and cortical injuries. Discharged 3 weeks afterwards to care facility on mechanical ventilation
Paul 2003 [10]	TSA for partial resection of pituitary tumor	TSA for partial resection of pituitary adenoma. During transcranial completion two weeks later, nasotracheal intubation was attempted. Encountered rapid pulsatile bleeding upon advancement. Bilateral fixed and dilated pupils 25 minutes after event. CT with blood in the third/fourth ventricles, basal cisterns, and around brainstem.	Nasotracheal tube	14	†	EEG showed minimal spontaneous activity and severe brainstem disturbance. Died 4 days later
Vahid 2007 [11]	TSA for resection of cranial base chordoma	Admitted to ICU 3 months postoperatively with multilobar pneumonia, intubated with NGT but developed quadriplegia soon after placement. CT demonstrated NGT in brainstem and spinal cord	10F NGT	90	†	Quadriplegic
Hanna 2012 [12]	Revision TSA for extended transclival resection of clival chordoma	NGT dislodged overnight and replaced not under direct visualization. Patient developed left-sided weakness with CT showing tube entering the brainstem and spinal cord	Small-bore feeding tube	†	Surgical site	Quadriplegic. Family withdrew care after 7 months.
Owens 2014 [13]	Revision TSA for resection of pituitary tumor	Developed suprasellar hemorrhage requiring ventriculostomy tube. Postop course thereafter included multiple re-intubations. Transnasal suctioning on POD25 with bloody secretions followed by decreased LOC with subsequent reintubation. CT showed pneumocephalus and new parenchymal hemorrhage in the L parietal region.	Nasotracheal suction catheter	25	†	Tracheostomy and feeding tube placed, discharged to care facility. Multiple medical complications, died 1 year after surgery
Zhang 2019 [14]	TSA for resection of pituitary tumor	Presented to ER with epistaxis after outside hospital placement of NGT. History of TSA for resection of pituitary adenoma 3 months prior. CT with intracranial tube and brain contusion, no intracranial vascular disruption on CTA.	NGT	90	†	Broad-spectrum antibiotics x3 weeks and discharged
Iglesias 2021 [15]	Transoral transsphenoidal resection of pituitary tumor	Past medical history of seizures, entriculoperitoneal shunt. Persistent seizures and failure to extubate requiring tracheostomy POD5. NGT inserted POD12 for poor orogastric tolerance. Post insertion XR showed shunt tubing that was mistaken for NGT. Developed uncontrollable seizures after insertion. CT showed tetraventricular hemorrhage with tubular structure passing through the sphenoid sinus into the third ventricle and onto the lateral ventricles	NGT	12	Sella	Refractory increased ICP and progressive general neurologic impairment. Family elected for comfort care and patient died 8 days after insertion
Obiorah 2020 [16]	Elective septoplasty	Re-intubated postoperatively and CT demonstrated right frontal ICH with IVH. Packing removed and NGT placed in ICU, transferred to larger institution. Repeat CT showed intracranial NGT.	NGT	0	Posterior cribriform and planum sphenoidale	Slow improvement, extubated day 5. Started having fevers and neurologic decline, TCD on day 8 showed mod-severe vasospasm. MRI w/ brainstem and bilateral hemisphere acute ischemic infarcts. Died on day 12
Nathoo (1999) [17]	Unilateral choanal atresia repair	Developed CSF leak managed conservatively and meningitis with CT showing basisphenoid defect. NGT placed POD14. Skull XR showed intracranial NGT, CT showing trajectory through basi-sphenoid region through basal cisterns, entering third ventricle and passing through pineal region into posterior interhemispheric cistern	NGT	14	Basisphenoid defect	Met appropriate milestones, no CSF rhinorrhea. CT at 9 months showed closed bony defect

Etiology and timing of intracranial tube malposition

Intracranial penetration by nasally introduced tubes remains an extremely rare occurrence, but the risk is increased when a skull base defect exists. Such a defect is most commonly encountered due to a traumatic fracture. In this systematic review, 62 cases were encountered, of which 36 (58%) were traumatic. Conversely, only 11 cases (18%) of post-surgical intracranial insertion were found, including the case described in this report. Additionally, this risk persists over time. Two of the published cases occurred three months post-surgery, and the case detailed here took place six months after initial skull base surgery.

Risk after sinonasal and skull base procedures

Although the majority of cases in this review occurred after transsphenoidal or transclival surgery, two cases were seen after other otolaryngologic surgeries. During septoplasty, twisting of the posterior bony septum to remove a portion of the perpendicular plate of the ethmoid bone can lead to fracture and/or defect along the cribriform plate [18]. The skull base is also at risk during choanal atresia repair due to the close proximity of the clivus and basisphenoid to the choana [19]. These cases highlight the need for caution after procedures during which the skull base can be damaged.

Preventative strategies: Intraoperative and postoperative

**Figure 3:**
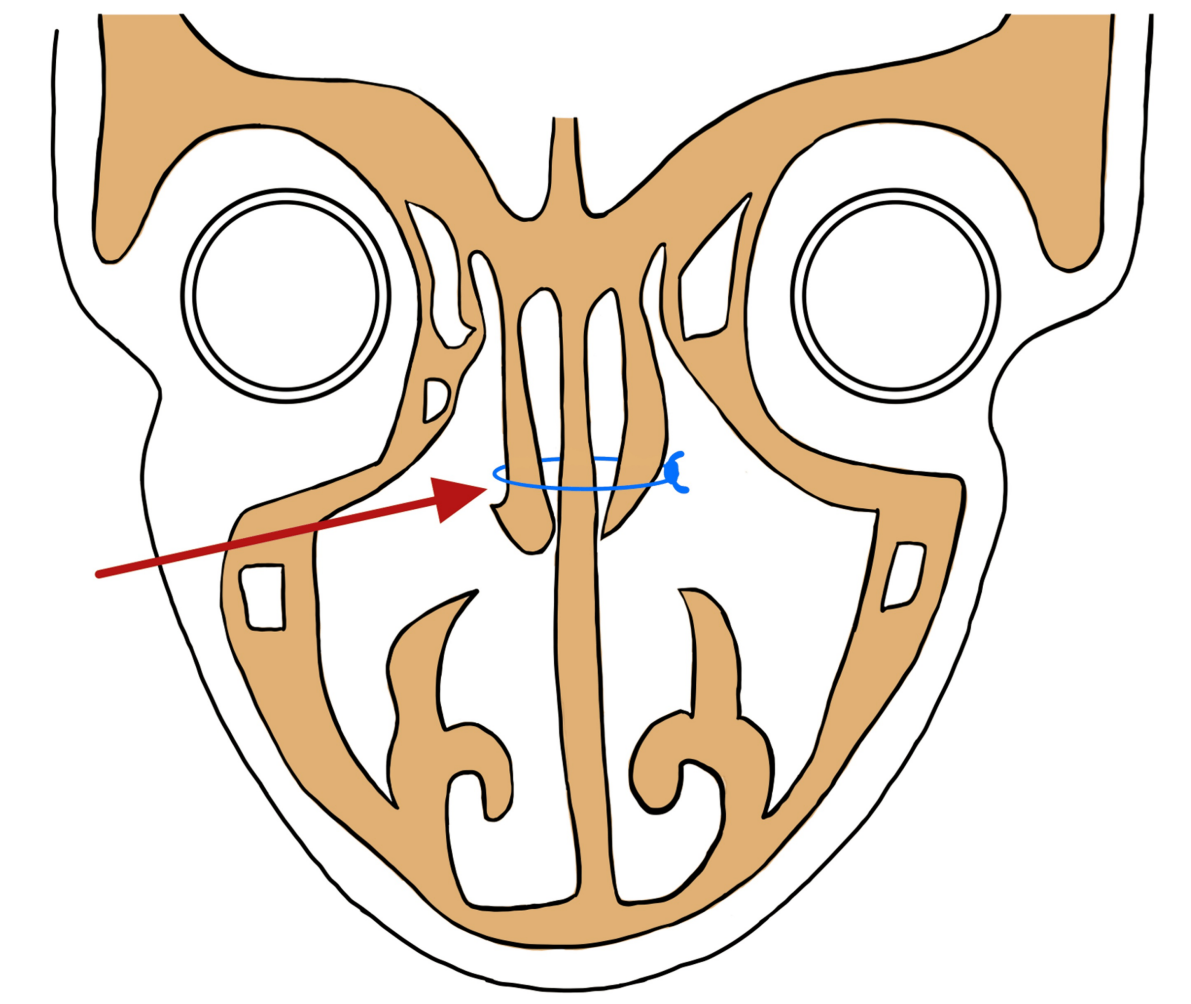
Coronal schematic showing medialization of middle turbinates with a red arrow. Technique for medialization is at the discretion of the surgeon, but suture marsupialization (blue), as displayed in this schematic, is one option. The figure was created by the author (ZN).

Several studies have attempted to find strategies to reduce the risk of intracranial penetration after skull base surgery. Bhattacharyya et al. conducted a cadaver study in which they performed standard endoscopic sinus surgery and deliberately attempted to contact the fovea ethmoidalis and sphenoid sinus with 16F and 18F NGT [20]. They found that the NGT entered the sphenoid sinus easily in 91.7% of specimens and could be preferentially funneled into the sinus by inferior septal spurs. However, they were not able to penetrate the sphenoid roof in any cases and thus concluded that the risk of intracranial penetration by NGT after standard endoscopic sinus surgery was low. However, this risk assessment changes in the context of transsphenoidal surgery, where a bony defect is deliberately created, potentially increasing the risk of intracranial penetration by NGT. Gill et al. describe several operative techniques to limit exposure of the sphenoid sinus, including wide unilateral sphenoidotomy with conservative contralateral sphenoidotomy, middle turbinate preservation and medialization, limited posterior septectomy, and transseptal approach to the sphenoid ostium [21]. Shah et al. used cadavers to perform a standard endoscopic transsphenoidal hypophysectomy. They studied the position of the middle turbinate in preventing sphenoid entry of the 10F Dobhoff tube and 16F NGT blindly placed by providers of different training levels [22]. They found that permanent medialization of the middle turbinate via septal pexy was superior to either neutral positioning or resection of the middle turbinate (Figure 3). They also found higher rates of entry with NGT as compared to the Dobhoff tube and hypothesized that this is due to the stiffer construction of NGT.

These aforementioned intraoperative tactics to protect the skull base (limited posterior septectomy, permanent medialization of the middle turbinate) can be considered; however, they must be weighed against the necessary operative exposure for safe and complete surgery, and the feasibility of postoperative visualization and care of the surgical site. There is insufficient data to conclude if a different repair strategy may provide extra protection in the case of sphenoid entry, which, as noted in the above study, is common. Postoperatively, we recommend the use of small-bore flexible feeding tubes over stiff NGT if possible. Authors have described the use of medical bracelets alerting providers that the patient has had a skull base procedure and to avoid blind instrumentation of the nose; however, data are needed to determine if this strategy is effective in terms of outcomes and cost [21]. We recommend educating patients and the involved family about the potential risks of subsequent nasal procedures pre- and postoperatively so that they may inform future providers.

Limitations of the literature

The reports included in this retrospective review are not without limitations. All reports included are single-case reports due to the rarity of this complication. Additionally, there is a lack of standardization when reporting the type or size of tube that was used. This limits the ability to make conclusions regarding whether a specific type of tube could be considered the highest risk in this patient population.

## Conclusions

Intracranial placement of nasally introduced tubes is a rare but devastating complication that can occur not only after traumatic skull base injuries but also following anterior skull base surgery. This case and systematic review highlight the critical importance of recognizing prior skull base or sinonasal procedures as a key risk factor for iatrogenic intracranial injury. Preventative strategies, including thoughtful surgical reconstruction, intraoperative techniques to minimize exposure, and the postoperative use of small-bore, flexible tubes, may reduce risk. However, clear guidelines remain lacking. Until more data are available, we advocate for heightened provider awareness, strong documentation of skull base surgical history, and direct patient and caregiver education regarding the lifelong risk of blind nasal instrumentation.
